# The Assessment of Cooking Skills and Food Skills and Their Relationship with Nutrition Knowledge, Attitude toward a Healthy Diet and Food Intake: Results of a German Validation Study

**DOI:** 10.3390/nu14153157

**Published:** 2022-07-30

**Authors:** Sonja Mötteli, Florian Hotzy

**Affiliations:** 1 Department of Psychiatry, Psychotherapy and Psychosomatics, Psychiatric University Hospital Zurich, 8032 Zurich, Switzerland; florian.hotzy@pukzh.ch; 2 Faculty of Medicine, University of Zurich, 8006 Zurich, Switzerland

**Keywords:** cooking skills, food skills, validation, reliability, food intake, healthy diet, nutrition knowledge, forward-backward translation

## Abstract

There is a lack of validated assessment instruments that capture all facets of cooking skills (CS) and food skills (FS). The goal of this study was to validate the German version of a questionnaire to assess a broad range of CS and FS and to examine its relationship with nutrition knowledge, attitude toward a healthy diet, and food intake. The German version was developed using forward–backward translation. An online survey was completed by students (*n* = 141), participants from the general Swiss population (*n* = 50), and nutrition experts (*n* = 18), including the CS and FS items along with nutrition knowledge, food frequency items, attitude toward a healthy diet and sociodemographic variables. The reliability and construct validity were examined. Results: For all of the samples, Cronbach’s alpha was between 0.85 and 0.88 for CS items and between 0.84 and 0.86 for FS items. The scales were strongly correlated (r = 0.60–0.77, *p* < 0.01). Nutrition experts showed higher confidence in their CS and FS than students and the participants of the general Swiss population (*p* < 0.001). CS and FS correlated weakly to moderately with practical nutrition knowledge, attitude toward a healthy diet, and the diet quality index. The German version is an efficient, valid, and highly reliable instrument that seems sensitive to changes. FS, compared to CS, might be more important for a healthy diet.

## 1. Introduction

A healthy and balanced diet requires a set of varied skills pertinent to the planning and management of meals and the selection and preparation of foods [1]. Because food preparation at home and eating homemade meals have been linked to better diet quality in both adults and children [2,3,4,5,6,7], interventions to improve the cooking skills (CS) and food skills (FS) of individuals have become popular in public health [8]. Although most of these interventions were positively related to dietary intake measures [8,9,10], there is a strong need for improved study designs and the use of validated assessment instruments that capture all facets of CS and FS [8,10]. Previous instruments often include a mix of CS, FS, and nutrition knowledge items and mention specific foods related to certain cultures [8].

To overcome the limitations of existing tools, Lavelle and colleagues (2017) developed two separate and comprehensive measures of CS and FS, taking the literature and expert opinions into account [11]. In a recent review, the frequency and type of cooking and food preparation and cooking confidence were identified as the main components of CS. Similarly, the planning, frequency, type of shopping behavior, food safety, and hygiene knowledge were identified as the main components of FS [8]. Therefore, in the new tools, CS includes different mechanical cooking and food preparation techniques (e.g., chopping and boiling), and FS includes a range of knowledge and skills necessary for preparing needs-based meals (e.g., planning and shopping) [11]. The scales demonstrated good psychometric properties and are user-friendly with simple task descriptions so that they can be used in different populations [11]. In a further study, the CS and FS scales were positively correlated with diet quality in Australian adults [12]. However, the validity of these measures and their generalisability to non-English-speaking populations needs to be investigated [11]. Therefore, the aim of this study was to conduct a validation study of the described CS and FS measures for German-speaking countries using a sample of students, a sample of adults from the general Swiss population, and a sample of nutrition experts.

## 2. Materials and Methods

This study is based on a cross-sectional online questionnaire conducted on three different samples. First (Sample 1), the German version of the measures assessing CS and FS and other variables such as nutrition knowledge, a proxy of diet quality, and sociodemographic characteristics were completed by a sample of students. Second (Sample 2), the measures assessing CS and FS were administered to a sample of adults of the general Swiss population, and third (Sample 3), to a sample of experts. In all of the samples, the participants rated their frequency of use and confidence in skills on a scale ranging from 0 to 7 (0 = never/rarely do it, 1 = very poor to 7 = very good). All of the data were assessed or entered fully anonymously in Limesurvey. Therefore, ethical approval was not required, as confirmed by the Ethics Committee of the Canton Zurich, Switzerland (Req-2022-00715).

### 2.1. Translation Process

The German version of the CS and FS scales was created using the forward and backward translation approach [13]. The first step was a forward translation of the original questionnaire from English to German by a native German speaker who worked in an English-speaking country at the time. The translator paid particular attention to conceptual equivalence. The translation was then discussed with the principal investigator and revised to produce a final version. A second independent and bilingual individual translated the German version of the questionnaire back to English. All of the items in the backward translation were discussed with the first author of the original questionnaire, and some modifications were made. The German version was then pilot tested for clarity and feasibility on five healthcare workers.

### 2.2. Sample 1

In November 2021, a sample of Psychology students from the Zurich University of Applied Sciences in Switzerland completed an online questionnaire including the CS and FS measures (see Table 1). In addition, they completed a control question asking whether their skills had changed in relation to the COVID-19 pandemic to be able to discriminate possible effects when comparing the results to the original questionnaire. The questionnaire also included the PKB-7 scale, measuring practical nutrition knowledge about balanced meals [14] and 16 simple and validated food frequency questions [14,15]. For instance, the participants were asked how often they eat vegetables (daily, 4–6x/week, 1–3x/week, 1–3x/month, seldom) and how many portions they usually eat (1–6 handfuls). Based on these items, we calculated a diet quality index as a proxy for a more or less healthy/unhealthy diet [15]. In addition, attitudes towards a healthy diet on a scale ranging from 1 = not important at all to 10 = very important, and sociodemographic variables (gender, age, living with parents or alone/with others) were assessed. All of the participants gave informed consent to participate. They did not receive financial compensation but were given 0.25 student credits, of which they should have 10 completed within two years. Among the 164 participants, individuals with response times <5 min and those with impossible answers (*n* = 23) were excluded. The final sample (*n* = 141) comprised 80% women, 19% men, and 1% not defined. The mean age was 28.0 years (SD = 8.0, range = 20–56), and 21% lived with their parents.

### 2.3. Sample 2

During September 2021 and June 2022, a sample of adults aged between 18 and 65 years from the general Swiss population was interviewed on the CS and FS items along with other measures described in Section 2.2 such as the PKB-7 scale [14], food frequency questions [14,15], and attitudes towards a healthy diet. In addition, gender, age, education level, and a question related to the COVID-19 pandemic were assessed. The participants were recruited using community-based recruitment strategies, such as advertisements and flyers posted in public places (e.g., working and healthcare institutions) and by snowball sampling. For this study, the first 25 male and the first 25 female participants were included. All of the participants provided written informed consent to participate and did not receive financial compensation. The sample (*n* = 50) comprised 50% women, the mean age was 37.8 years (SD = 11.6, range = 21–62) and 38% had a university degree.

### 2.4. Sample 3

In addition to the non-expert samples, the CS and FS items were completed via an online survey by 18 dieticians from the University Hospital Zurich in Switzerland between July and August 2021. This expert sample comprised 89% women and 11% men with a mean age of 35.2 years (SD = 9.5, range = 24–63). All of the participants gave informed consent to participate and did not receive any financial compensation.

### 2.5. Statistical Analyses

First, the scores on each scale were summed up. The internal consistency reliability of each scale was determined using Cronbach’s alpha. Discriminant validity was tested by comparing the scores of experts with high levels of CS and FS with those of laypersons using *t*-tests for independent samples if homogeneity of variance was given. According to sample size calculation with alpha = 0.025, 1-beta = 80 and effect size d = 1.0, 18 experts should be sufficient to find significant differences. In addition, correlation coefficients were calculated between CS and FS and related variables such as nutrition knowledge, diet quality index, and attitude towards a healthy diet. There were no missing variables. The statistical analyses were conducted using SPSS version 28 with significance levels set to 5%. For directed hypothesis-testing, the significance level was set to 2.5%.

**Table 1:** Usage and confidence in cooking skills and food skills.

	Sample 1: Students (*n* = 141)				Sample 2: Adults from the Swiss General Population (*n* = 50)				Sample 3: Dieticians (*n* = 18)			
	Usage		Confidence (Rated 1–7)		Usage		Confidence (Rated 1–7)		Usage		Confidence (Rated 1–7)	
**Cooking skills**	*n*	*%*	*M*	*SD*	*n*	*%*	*M*	*SD*	*n*	*%*	*M*	*SD*
1. Chop, mix, and stir foods	141	100.0	5.81	1.13	50	100.0	5.76	1.30	18	100.0	6.83	0.51
2. Blend foods to make them smooth, such as soups or sauces	128	90.8	5.42	2.09	40	80.0	4.60	2.79	18	100.0	6.72	0.96
3. Steam food	126	89.4	4.50	2.21	41	82.0	3.82	2.55	18	100.0	6.89	0.32
4. Boil or simmer food	140	99.3	5.78	1.25	47	94.0	5.08	2.09	18	100.0	6.72	0.57
5. Stew food	100	70.9	3.09	2.50	35	70.0	3.28	2.88	15	83.3	5.39	2.59
6. Roast food in the oven	136	96.5	4.91	1.75	41	82.0	4.28	2.56	17	94.4	6.61	0.61
7. Fry/stirfry food in a frying pan/wok with oil or fat	135	95.7	5.82	1.14	40	80.0	4.98	2.16	17	94.4	6.50	0.86
8. Microwave food	125	88.7	5.12	2.39	28	56.0	3.00	3.05	13	72.2	4.61	3.09
9. Bake goods	129	91.5	4.72	2.13	25	50.0	2.42	2.73	17	94.4	6.50	1.65
10. Peel and chop vegetables	140	99.3	5.82	1.43	49	98.0	5.74	1.64	17	94.4	6.72	0.57
11. Prepare and cook raw meat/poultry	96	68.1	3.12	2.64	43	86.0	4.36	2.52	15	83.3	5.61	2.62
12. Prepare and cook raw fish	80	56.7	2.30	2.47	39	78.0	3.92	2.72	15	83.3	4.89	2.42
13. Make sauces and gravy from scratch	115	81.6	3.39	2.28	49	98.0	3.04	2.63	17	94.4	5.11	1.78
14. Use herbs and spices	139	98.6	5.64	1.38	50	100.0	5.44	1.73	17	94.5	6.83	0.51
Overall cooking skills score			65.43	16.04			59.72	20.55			85.94	14.41
**Food skills**												
1. Plan meals ahead?	135	94.6	4.35	1.76	39	78.0	3.54	2.67	17	94.4	5.61	1.91
2. Prepare meals in advance?	130	92.2	4.31	1.89	32	64.0	2.68	2.63	18	100.0	6.00	0.97
3. Follow recipes when cooking?	134	95.0	5.05	1.86	41	82.0	4.00	2.53	15	83.3	5.39	2.62
4. Shop with a grocery list?	137	97.2	5.70	1.66	39	78.0	4.06	2.75	17	94.4	6.00	1.85
5. Shop with specific meals in mind?	137	97.2	5.07	1.78	45	90.0	4.46	2.26	14	77.8	6.39	0.85
6. Plan how much food to buy?	137	97.2	4.54	1.83	46	92.0	4.24	2.39	16	88.9	6.22	1.06
7. Compare prices before you buy food?	117	83.0	3.70	2.38	37	74.0	3.34	2.68	13	72.2	4.44	2.96
8. Know what budget you have to spend on food?	130	92.2	4.11	2.15	41	82.0	3.84	2.45	16	88.9	4.61	2.35
9. Buy food in season to save money?	135	95.7	4.42	1.78	38	76.0	3.38	2.49	17	94.4	6.00	1.64
10. Buy cheaper cuts of meat to save money?	63	44.7	2.05	2.59	23	46.0	1.78	2.30	13	72.2	3.83	2.98
11. Cook more or double recipes which can be used for another meal?	130	92.2	5.13	1.98	38	76.0	3.80	2.73	15	83.3	6.28	0.75
12. Prepare or cook a healthy meal with only few ingredients on hand?	139	98.6	5.42	1.54	47	94.0	4.96	1.96	16	88.9	6.39	0.70
13. Prepare or cook a meal with limited time?	140	99.3	5.50	1.45	48	96.0	5.48	1.81	14	77.8	6.39	0.85
14. Use leftovers to create another meal?	138	97.9	5.44	1.66	44	88.0	4.80	2.25	16	88.9	6.44	0.70
15. Keep basic items in your cupboard for putting meals together?	137	97.2	5.22	1.71	44	88.0	4.78	2.29	16	88.9	5.89	1.37
16. Read the best-before date on food?	135	95.7	5.16	1.91	47	94.0	5.66	2.01	17	94.4	5.83	1.86
17. Read the storage and use-by information on food packets?	132	93.6	4.90	2.15	34	68.0	3.64	2.96	16	88.9	5.78	2.24
18. Read the nutrition information on food labels?	117	83.0	3.93	2.53	31	62.0	3.24	2.99	17	94.4	6.56	0.78
19. Balance meals based on nutrition advice on what is healthy?	117	83.0	3.91	2.41	35	70.0	2.76	2.57	14	77.8	6.78	0.43
Overall food skills score			87.92	19.79			74.44	25.22			110.83	16.35

## 3. Results

### 3.1. Usage Rate and Confidence in Cooking Skills and Food Skills

Most of the study participants of all samples reported regularly using the different CS and FS. Their usage rate and level of confidence in these skills are presented in Table 1. Mean confidence levels of CS and FS were lowest in the sample of the general Swiss population. For this sample (*n* = 50), female participants had significantly superior CS (M = 70.68, SD = 17.65) and FS (M = 85.12, SD = 24.43) compared to male participants (CS: M = 48.76, SD = 17.34, *p* < 0.001; FS: M = 63.76, SD = 21.54, *p* = 0.002). Age significantly correlated with CS but not FS (r_s_ = 0.29, *p* = 0.044; r_s_ = 0.18, *p* = 0.223 *n* = 50). Education level did not significantly correlate with CS but with FS (r_s_ = 0.14, *p* = 0.328; r_s_ = 0.32, *p* = 0.024 *n* = 50).

A third of the student sample (n = 49, 34.8%) reported that their CS had improved due to the COVID-19 pandemic, whereas 92 (65.2%) indicated that there had been no change. Similarly, 38 students (27.0%) reported that their FS had improved, 102 students (72.3%) reported no change, and one student (0.7%) reported a deterioration in FS. In the adult sample of the general Swiss population, 80% reported no effects on dietary behavior.

The median response time for items on the CS and FS scales was 3.4 min.

### 3.2. Reliability and Validity

The Cronbach’s alpha (internal consistency reliability) of the CS items was 0.85 for the student sample (*n* = 141), 0.87 for the sample of adults from the general Swiss population (*n* = 50), and 0.88 for the expert sample (*n* = 18). Cronbach’s alpha of the FS items was 0.86 for the student sample, 0.86 for the sample of adults from the general Swiss population, and 0.84 for the expert sample. The CS were strongly correlated with the FS in all samples (students: r = 0.60, *p* < 0.001; general Swiss population: r = 0.77, *p* < 0.001; nutrition experts: r = 0.66, *p* = 0.003), which confirmed that the scales measure highly related constructs.

A significantly higher level of confidence in CS was reported in the expert sample (M = 85.9, SD = 14.4) than in the student sample (M = 65.4, SD = 16.0, t (157) = 5.2, *p* < 0.001, Cohen’s d =1.35). The level of FS reported in the expert sample (M = 110.8, SD = 16.4) was also significantly higher than in the student sample (M = 87.9, SD = 19.8, t (157) = 4.7, *p* < 0.001, Cohen’s d =1.26).

Furthermore, the student participants who lived alone or with individuals other than their parents (*n* = 111) reported significantly higher CS (M = 67.7, SD = 15.1) compared to the 30 students who still lived with their parents (M = 56.9, SD = 16.7, t (139) = 3.4, *p* < 0.001, Cohen’s d = 0.68). However, there was no significant difference in FS between these subgroups (M = 88.3, SD = 20.4 versus M = 86.4, SD = 17.6, t (139) = 0.5, *p* = 0.32).

### 3.3. Associations with Nutrition Knowledge, Attitude toward a Healthy Diet and Food Intake

To determine the associations between CS and FS, the total number of participants from Samples 1 and 2 was used (*n* = 191). Table 2 shows the correlation matrix. Higher levels of CS and FS positively correlated with practical nutrition knowledge, but only significantly for FS. The participants with higher levels of CS and FS rated a healthy diet as more important and had a higher diet quality index. FS still correlated with the diet quality index if controlled for CS and nutrition knowledge (r = 0.24, *p* < 0.001), but CS did no longer correlate with the diet quality index if controlled for FS and nutrition knowledge (r = −0.03, *p* = 710).

## 4. Discussion

In this study, two recently developed measures for assessing confidence in CS and FS were translated and validated for German-speaking countries. The mean values of the students’ CS and FS (M = 65.4, SD = 16.0 and M = 87.9, SD = 19.8) were very similar to those of the student sample in the original study [11] (M = 58.8, SD = 16.5 and M = 74.8, SD = 27.6), considering the indicated improvement in these skills during the COVID-19 pandemic. This indicates the validity of the translated version and the good applicability of the measures. The frequency of use and the mean values of CS and FS in the adult sample of the general Swiss population (M = 59.7, SD = 20.6 and M = 74.4, SD = 25.2) were much higher compared to those of the nationally representative sample in the original study (M = 47.8, SD = 29.3 and M = 45.8, SD = 38.6). The reasons for that might be the small sample size and higher educational background of our sample (38% with a university degree compared to the average Swiss population with 30%) and perhaps the COVID-19 pandemic-induced “learning boost” regarding CS and FS. Nevertheless, the majority stated that the COVID-19 pandemic had no impact on their CS and FS. Another reason for higher CS and FS in the Swiss participants might be that the variability and consumption of convenience food in English-speaking countries is very high, while a higher intake is associated with lower CS and FS [10]. In general, the high frequency of usage rates (range 44.7–100%) on different scale items shows that the described tasks capture the relevant CS and FS. In all samples, the frequency of using simple cooking tasks such as peeling, chopping, and boiling food was higher compared to more advanced cooking tasks such as stewing food. In the student sample, and in comparison to the adult sample of the general Swiss population, the usage rate for skills related to the preparation of raw meat and fish was lower than for other items, such as preparing vegetables or baking goods, as known for predominantly female samples [16].

The Cronbach’s Alpha for the CS and FS items indicates that both scales were highly reliable. Furthermore, this study shows discriminate validity as the nutrition experts (dieticians) had significantly higher confidence levels in their CS and FS compared to laypeople. In keeping with the results of the original study [11], the scales were able to distinguish between higher skilled and lower skilled participants.

In addition, CS and FS were substantially correlated with a positive attitude toward a healthy diet, which indicates the importance of nutrition-related health education. Further, the results show that FS, as more cognitive tasks, seem to be closer linked to nutrition knowledge than CS, as more mechanical tasks. In line with a previous study, FS were higher correlated with diet quality compared to CS [12]. Therefore, FS seem to be more important for eating a healthy diet than cooking skills, particularly nowadays with the possibility of choosing a variety of different pre-prepared foods.

In summary, the results show that the German version of the measures assessing confidence levels in CS and FS is valid and highly reliable. Due to the measures’ simple wording and efficiency, the scales can be used as screening or monitoring tools for the assessment of CS and FS in research and also in nutrition counseling and clinical settings, including populations with different socio-economic backgrounds. They are easy to complete and might help to discriminate whether diet-related problems (such as metabolic syndrome, malnutrition, and others) are based on a lack of CS and FS or if other factors have to be taken into account to achieve a balanced diet. Beyond the results of scale validation, this study emphasizes the importance of practical knowledge such as food skills for adopting a healthy diet.

## Figures and Tables

**Table 2:** Associations with cooking skills and food skills.

Variables	Possible Range	1	2	3	4	5
1. Cooking skills (CS)	0 to 98 (higher scores = more confidence in CS)	1	0.67 **	0.13	0.38 **	0.19 *
2. Food skills (FS)	0 to 133 (higher scores = more confidence in FS)		1	0.19 *	0.34 **	0.31 **
3. Practical nutrition knowledge	0 to 7 (higher scores = more knowledge)			1	0.15 *	0.09
4. Attitude toward a healthy diet	1 to 10 (higher scores = healthy diet is more important)				1	0.34 **
5. Diet quality index	0 to 6 (higher scores = better diet quality)					1
M		63.94	84.39	5.3	8.04	3.07
SD		17.46	22.09	1.31	1.53	1.49

Notes. N = 191 including Sample 1 and Sample 2; Pearson-Correlations; ** *p* < 0.001, * *p* < 0.05.

## Data Availability

The dataset based on this study is available upon request to the authors.
